# Crystal polymorphism in fragment-based lead discovery of ligands of the catalytic domain of UGGT, the glycoprotein folding quality control checkpoint

**DOI:** 10.3389/fmolb.2022.960248

**Published:** 2022-12-14

**Authors:** Alessandro T. Caputo, Roberta Ibba, James D. Le Cornu, Benoit Darlot, Mario Hensen, Colette B. Lipp, Gabriele Marcianò, Snežana Vasiljević, Nicole Zitzmann, Pietro Roversi

**Affiliations:** ^1^ Biochemistry Department, Oxford Glycobiology Institute, University of Oxford, Oxford, United Kingdom; ^2^ Commonwealth Scientific and Industrial Research Organisation, Clayton, VIC, Australia; ^3^ Department of Medicine, Surgery and Pharmacy, University of Sassari, Sassari, Italy; ^4^ Wellcome Trust Centre for Cell Biology, University of Edinburgh, Scotland, United Kingdom; ^5^ Biochemistry Department, University of Oxford, Oxford, United Kingdom; ^6^ IBBA-CNR Unit of Milano, Institute of Agricultural Biology and Biotechnology, Milano, Italy; ^7^ Department of Molecular and Cell Biology, Leicester Institute of Structural and Chemical Biology, University of Leicester, Leicester, United Kingdom

**Keywords:** UGGT, crystal polymorphism, structure-based lead discovery, structure determination pipeline, [(morpholin-4yl)methyl]quinolin-8-ol

## Abstract

None of the current data processing pipelines for X-ray crystallography fragment-based lead discovery (FBLD) consults all the information available when deciding on the lattice and symmetry (i.e., the polymorph) of each soaked crystal. Often, X-ray crystallography FBLD pipelines either choose the polymorph based on cell volume and point-group symmetry of the X-ray diffraction data or leave polymorph attribution to manual intervention on the part of the user. Thus, when the FBLD crystals belong to more than one crystal polymorph, the discovery pipeline can be plagued by space group ambiguity, especially if the polymorphs at hand are variations of the same lattice and, therefore, difficult to tell apart from their morphology and/or their apparent crystal lattices and point groups. In the course of a fragment-based lead discovery effort aimed at finding ligands of the catalytic domain of UDP–glucose glycoprotein glucosyltransferase (UGGT), we encountered a mixture of trigonal crystals and pseudotrigonal triclinic crystals—with the two lattices closely related. In order to resolve that polymorphism ambiguity, we have written and described here a series of Unix shell scripts called *CoALLA* (*c*rystal p*o*lymorph *a*nd *l*igand *l*ikelihood-based *a*ssignment). The *CoALLA* scripts are written in Unix shell and use *autoPROC* for data processing, *CCP4-Dimple*/*REFMAC5* and *BUSTER* for refinement, and *RHOFIT* for ligand docking. The choice of the polymorph is effected by carrying out (in each of the known polymorphs) the tasks of diffraction data indexing, integration, scaling, and structural refinement. The most likely polymorph is then chosen as the one with the best structure refinement R_free_ statistic. The *CoALLA* scripts further implement a likelihood-based ligand assignment strategy, starting with macromolecular refinement and automated water addition, followed by removal of the water molecules that appear to be fitting ligand density, and a final round of refinement after random perturbation of the refined macromolecular model, in order to obtain unbiased difference density maps for automated ligand placement. We illustrate the use of *CoALLA* to discriminate between H3 and P1 crystals used for an FBLD effort to find fragments binding to the catalytic domain of *Chaetomium thermophilum* UGGT.

## 1 Introduction

There is an urgent medical need to develop novel antiviral drugs, as exemplified by the recent Ebola, Zika, and SARS-CoV-2 outbreaks (Pardi and Weissman, 2020). In particular, an FDA-approved host-targeting broad-spectrum antiviral could revolutionize the treatment of existing and newly emerging viruses (Dwek et al., 2022). Recent works elucidating the structures of key endoplasmic reticulum (ER) enzymes assisting the folding of viral glycoproteins have opened new avenues for identifying novel antivirals (Caputo et al., 2016; Roversi et al., 2017; Warfield et al., 2020).

Host-targeting broad-spectrum antivirals are a possibility because many viruses hijack the same host enzymes during their life cycle (Oksenych and Kainov (2022). For example, the envelope glycoproteins of many viruses are exquisitely dependent on calnexin-mediated folding, a process enabled by the action of the ER endoplasmic reticulum enzyme UDP–Glc glycoprotein glucosyltransferase (UGGT) and the ER alpha glucosidases I and II (D’Alessio et al., 2010). ER alpha glucosidases I and II usher client proteins in and out of the calnexin cycle, which is part of the glycoprotein folding quality control in the ER, whilst UGGT plays a key role in retaining misfolded glycoproteins in the ER for a “second chance” at folding correctly (Hammond et al., 1994).

One of the more advanced strategies for host-targeting broad-spectrum antiviral drug development is focusing on iminosugars as active site inhibitors of the ER alpha glucosidases (Alonzi et al., 2017; Tyrrell et al., 2017). However, as carbohydrate mimics, iminosugar inhibitors of the ER alpha glucosidases have some undesired off-target effects, as they also inhibit certain other carbohydrate processing enzymes within the human host (Sayce et al., 2016). A new class of molecules inhibiting host glycoprotein folding enzymes that viruses depend upon would have great potential for antiviral therapy (Karade et al., 2021). To complement a programme of development of new allosteric ER alpha glucosidase inhibitors with fewer off-target effects, we endeavoured to investigate the potential of UGGT, the major calnexin cycle misfold sensor (Trombetta et al., 1989), as a novel antiviral target (Tax et al., 2019). Currently, no UGGT inhibitors are known other than the product UDP (Trombetta and Helenius, 1999) and the UDP–glucose analogue UDP-2-deoxy-2-fluoro-D-glucose (U2F), neither of which is selective for UGGT.

One effective strategy to broaden the knowledge of the chemical space of a given protein target is fragment-based lead discovery (FBLD), a sequence of experiments enabling the extraction of ligands of a chosen target macromolecule from a chemical library (Ciulli et al., 2006; Murray and Blundell, 2010; Chen et al., 2015; Müller et al., 2022). Whenever crystals of the target macromolecule reliably diffract to near-atomic resolution, single-crystal X-ray crystallography is one of the main techniques successfully used for FBLD (Ciulli et al., 2006; Murray and Blundell, 2010; Radoux et al., 2016; Müller et al., 2022).

We recently determined crystal structures of the ER glycoprotein folding quality control checkpoint enzyme, the UDP-Glc glycoprotein glucosyltransferase of *Chaetomium thermophilum* (*Ct*UGGT) (Roversi et al., 2017). As well as a potential drug target against viruses (Dalziel et al., 2014), UGGT could be a target for compounds rescuing slightly misfolded and yet active glycoprotein mutants in certain instances of congenital rare disease (Amara et al., 1992) and against some cancers (Tax et al., 2019). We set out to find small-molecule ligands for this target *via* X-ray crystallography FBLD.

**FIGURE 1:**
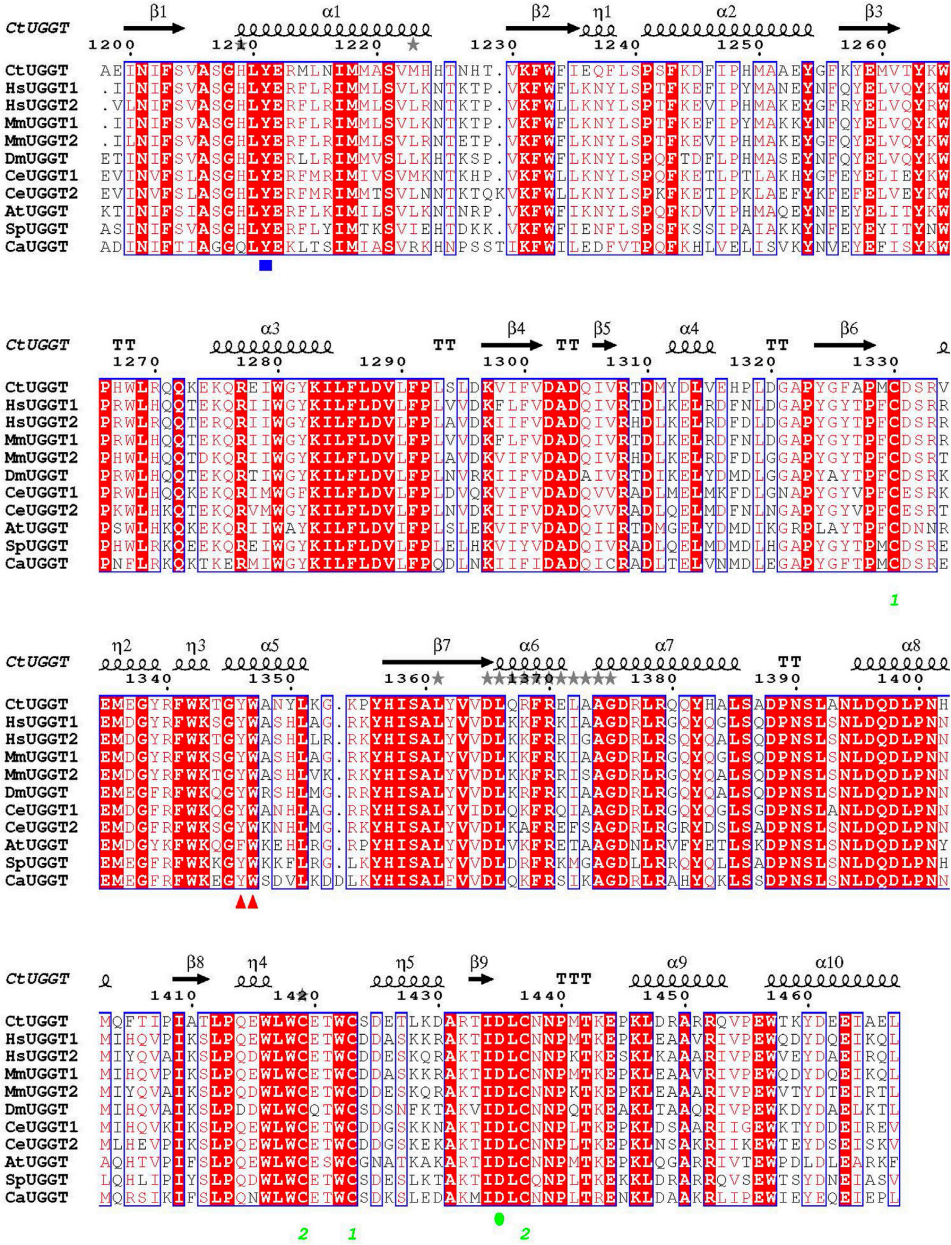
Sequence alignment of GT24 domains of a few eukaryotic UGGTs. *Ct: Chaetomium thermophilum*; *Hs: Homo sapiens*; *Mm: Mus musculus*; *At: Arabidopsis thaliana*; *Ce: Caenorhabditis elegans*; *Dm: Drosophila melanogaster*; *Sp: Schizosaccharomyces pombe*; *Ca: Candida albicans*. The *Ct*UGGT D1302 and D1304 residues coordinating the catalytic Ca^2+^ ion are completely conserved across these sequences. Red triangles mark the *Ct*UGGT ^1346^WY^1347^ clamp. A blue square marks the position of *Ct*UGGT Y1211 (coordinating the UDP–Glc uracyl ring). A green circle marks the position of *Ct*UGGT D1435 (coordinating the Ca^2+^ ion).

We could not crystallise the GT24 catalytic domain of human UGGT—so we used crystals of the GT24 catalytic domain of *Ct*UGGT (hereinafter CtUGGT_*GT*24_) instead. The sequence of this fungal UGGT has about 70% similarity and 60% identity to the ones of the same domain of the two human UGGT isoforms (UGGT1 and UGGT2, see Figure 1) so that any ligands found with the crystals of *Ct*UGGT would likely bind the human enzymes too—paving the way to a medicinal chemistry program towards modulators of human UGGT activity. In order to avoid fragments that would bind to the UDP–Glc pocket (and would then likely have some off-target affinity for a number of human glucosyltransferases using the same co-factor (Albesa-Jové and Guerin, 2016), the crystals used for the FBLD study were grown in the presence of Ca^2+^ and UDP–Glc (hereinafter *Ct*UGGT_GT24_^UDP−Glc^).

The *Ct*UGGT_GT24_^UDP−Glc^ crystals turned out to belong to two different polymorphs. Crystal polymorphism refers to the growth of crystals of the same compound belonging to different crystal forms. Each of the crystal forms for the same compound is referred to as one of its crystal polymorphs. All polymorphs contain the same molecule, but each polymorph has its own distinct crystal lattice and/or space group symmetry and/or asymmetric unit contents (Buerger, 1936a; Buerger, 1936b). A number of different polymorphs can grow from a few related crystallisation conditions—or even from crystallisation solutions that are nominally the same but experience slight stochastic variations in variables such as temperature, rate of evaporation, and impurities (Jurnak, 1985; Carter et al., 1994; Zabara et al., 2011; Yekwa et al., 2017).

The occurrence of crystal polymorphism during FBLD efforts is not uncommon. For example, in a recent crystallographic screening project, 364 diffraction datasets were collected each from a crystal individually soaked with one compound from a library; of these, 16 crystals belonged to the orthorhombic *P*2_1_2_1_2_1_ space group instead of the common monoclinic *P*2_1_ form; the two unit cells were also closely related (Schiebel et al., 2016). It is of note that systematic exploration of crystal polymorphism prior to FBLD can be of great advantage: the best diffracting polymorph can be selected, and various lattices expose different potential drug-binding sites (Vera et al., 2013). Unfortunately, implementation of polymorph assignment in automated FBLD data processing pipelines still leaves some to be desired.

For example, the *XCE* FBLD X-ray data processing pipeline used at Diamond Light Source (Krojer et al., 2017; Collins et al., 2018; Douangamath et al., 2021; Pearce et al., 2022) decides on the crystal polymorph using a comparison of point group symmetry and cell (ignoring the information encoded by the known polymorph atomic models), or it leaves polymorph attribution to manual intervention on the part of the user. The FBLD efforts at the BESSY and MAXIV synchrotrons use *FragMAXapp* for data processing (Lima et al., 2021); at EMBL Grenoble, the *CRIMS* suite is a large-scale, automated fragment screening pipeline enabling evaluation of libraries of over 1,000 fragments (Cornaciu et al., 2021); the IspyB system used at some synchrotrons allows for data integration and storage in alternative lattices in parallel, specifically to address the possibility of multiple polymorphs (Monaco et al., 2013); the FBLD efforts at the Swiss Light Source (SLS) rely on the *FFCS* processing pipeline (Kaminski et al., 2022). Regrettably, none of these FBLD data processing systems has a mechanism in place for automated polymorph assignment.

Overall, current implementations of the FBLD discovery process can be plagued by space group and cell ambiguity, especially if the polymorphs at hand are variations of the same lattice and, therefore, difficult to tell apart from their morphology and/or their apparent crystal lattices and point groups.

In order to expedite the analysis of the *Ct*UGGT_GT24_^UDP−Glc^ FBLD X-ray diffraction datasets, we have written and described here a series of Unix shell scripts called *CoALLA* (*c*rystal p*o*lymorph *a*nd *l*igand *l*ikelihood-based *A*ssignment). The *CoALLA* scripts are written in Unix shell and use *autoPROC* (Vonrhein et al., 2011) for data processing, *CCP4-Dimple*/*REFMAC5* (Murshudov et al., 2011; Winn et al., 2011; Keegan et al., 2015) and *BUSTER* (Blanc et al., 2004; Bricogne et al., 2017) for refinement, and *RHOFIT* (Vonrhein et al., 2011) for ligand placement.

Unique to *CoALLA* is the implementation of the choice of the polymorph, which is effected by carrying out diffraction data indexing, integration, scaling, and structural refinement in each of the possible polymorphs. The most likely polymorph is then chosen based on the best structure refinement statistics.

## 2 *Ct*UGGT_GT24_^UDP−Glc^ crystal structure

**FIGURE 2:**
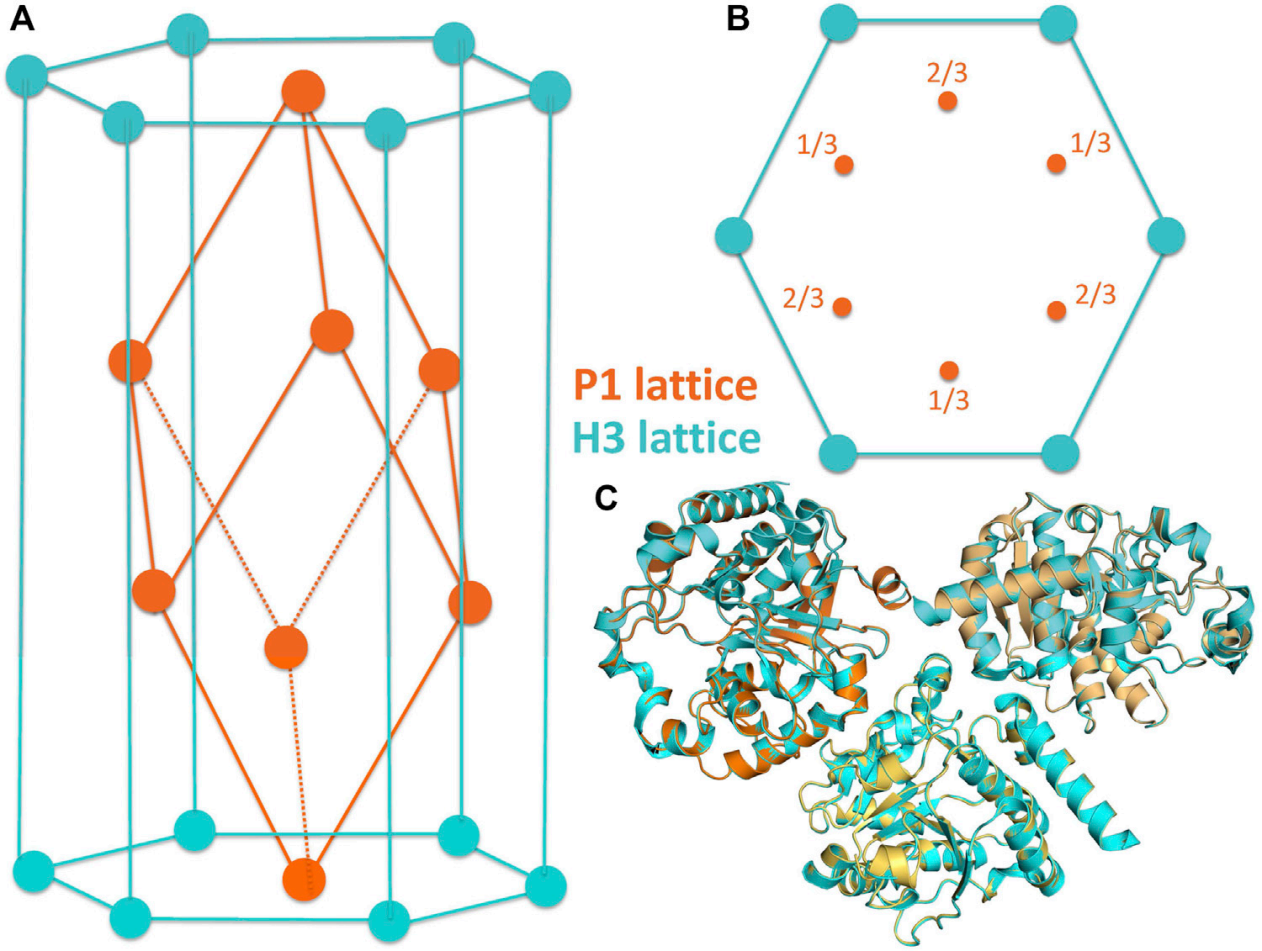
**(A,B)** Related crystal symmetries and lattices of the H3 (cyan) and P1 (orange) crystal forms of the *Ct*UGGT_*GT*24_ crystals. **(C)**The molecule in the asymmetric unit of the H3 crystal is shown, together with two of its symmetry mates (cyan), in cartoon representation. This portion of the H3 lattice has been overlaid onto the asymmetric unit of the P1 crystals (three chains, painted orange, light orange, and yellow-orange; also in cartoon representation) by superposing the “A” of the H3 crystal to the “A” chain in the P1 crystal.

**FIGURE 3:**
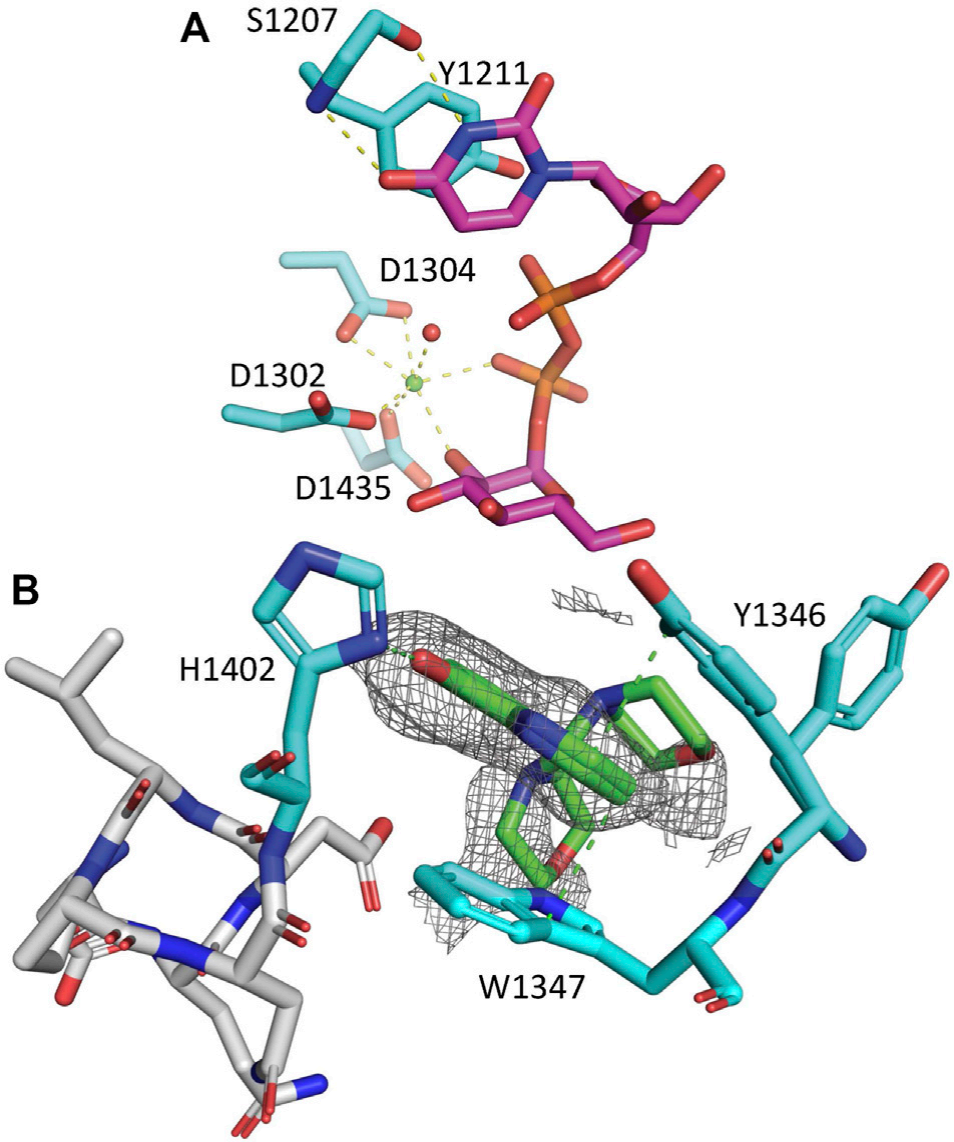
*Ct*UGGT_GT24_^UDP−Glc^  and *Ct*UGGT_GT24_^5M−8OH−Q^ crystal structures. **(A)** *Ct*UGGT_GT24_^UDP−Glc^ (PDB ID 6FSN). Protein atoms in stick representation; C cyan (but UDP–Glc C magenta), O red, N blue, P orange. H-bonds and Ca-coordination bonds are in yellow dashed lines. At the top, the residues coordinating the uracyl ring: the side chain of *Ct*UGGT Y1211 and the main chain of S1207. At the bottom, the Ca^2+^ ion is a green sphere, and its coordinating water molecules are red spheres. The side chains of residues D1302, D1304, and D1435 coordinate the Ca^2+^. Three more coordination sites are taken up by the *β* phosphate, the O2′ atom of its Glc ring, and a water molecule. The uracyl O4 atom accepts an H-bond from the S1207 main chain NH. **(B)** Zoom onto the *Ct*UGGT ^1346^YW^1347^ clamp (C atoms in cyan) binding 5M-8OH-Q (C atoms in green). The 8OH-quinoline ring inserts and is sandwiched between the aromatic side chains of the conserved residues ^1346^YW^1347^. The two aromatic side chains stabilise the quinoline ring, forming an aromatic trimer; the 8-OH group of the quinoline also establishes an H-bond to the side chain of ^1402^H. Representative distances to interacting residues are in green dashed lines. Only two of the many morpholine ring placements are shown. The unbiased Fo–Fc map is represented as a grey mesh at a 2.0 *σ* contour level. PDB ID: 7ZXW.

The crystals of *Ct*UGGT_GT24_^UDP−Glc^ used for the FBLD effort belonged to the space group H3 (with cell edges a = b = 118.8 Å  and c = 68.8 Å  (cyan cell in Figures 2A,B, PDB ID 6FSN)) with one molecule per asymmetric unit. The crystals likely capture a conformation of UDP–Glc following initial binding to the protein: the co-factor’s ribose ring points towards the solvent (Figure 3A). The uracyl ring O4 atom accepts a hydrogen bond from the main chain NH of S1207, and its N_3_ atom donates one hydrogen bond to the main chain O of the same residue (top of Figure 3A). Half of the coordination sphere of the Ca^2+^ ion in the *Ct*UGGT_*GT*24_ active site is occupied by the side chains from D1302 and D1304 belonging to the UGGT conserved ^1302^DAD^1304^ motif) and the side chain of the conserved D1435; the remaining three sites are occupied by an O atom from the *β* phosphate of UDP–Glc, the O2′ atom of the Glc ring, and a water molecule (Figure 3A).

### 2.1 Fragment-soaked *Ct*UGGT_GT24_^UDP−Glc^ crystal index in related P1 or H3 lattices

To discover *Ct*UGGT_*GT*24_ ligands by FBLD, 768 *Ct*UGGT_GT24_^UDP−Glc^ crystals were soaked with as many compounds, and X-ray diffraction datasets were collected from each soaked crystal (see Table 1). During the initial analysis of these X-ray diffraction datasets, we discovered that, upon soaking, crystal symmetry was sometimes lowered to P1, with three molecules in the asymmetric unit of a pseudo-rhombohedral primitive cell (orange cell in Figures 2A,B, PDB ID 7ZLU) of dimensions a = 66.3 Å, b = 72.7 Å, c = 72.7 Å, *α* = 111.2°, *β* = 107.7°, and *γ* = 107.7°. This cell is a distortion of the R3 primitive rhombohedral cell, which for the *Ct*UGGT_GT24_^UDP−Glc^ H3 crystals used for the soaks, would have lattice parameters a = b = c = 72.32 Å  and *α* = *β* = *γ* = 110.43°. The three molecules in the P1 crystal asymmetric unit are related by an NCS threefold axis equivalent to the H3 crystallographic threefold axis. The two packings are difficult to distinguish (see Figure 2C), and often a soaked *Ct*UGGT_GT24_^UDP−Glc^ X-ray diffraction dataset can index in both the H3 and P1 lattices, depending, for example, on the program used for indexing or the parameters of the automated indexing algorithm.

**TABLE 1:** Summary of the FBLD effort on *Ct*UGGT_*GT*24_.

Crystallisation experiments (sitting drops)	∼ 1,800
Soak experiments	768
Soaked crystals mounted/cryoprotected	692
Diffraction experiments	732
Processed diffraction datasets (H3-only, P1-only, or both)	493 (266, 25, 202)
Successfully refined (lower R_free_ in H3 and lower R_free_ in P1)	439 (390, 49)
Fragments bound	3

More than one dataset was collected from some crystals. A total of 96 crystals were soaked and mounted/cryoprotected but were not irradiated due to a mistake in loading the sample changer.

The *Ct*UGGT_*GT*24_ FBLD datasets were initially processed through the *XCE* workflow tool (Krojer et al., 2017). Space group assignment in the *XCE* workflow relies on automated data processing taking place during automated data collection on the Diamond I04-1 beamline (Douangamath et al., 2021), but the decision about the possible space group and cell of a certain dataset is made without consulting available models. In essence, the initial *XCE* automated decision regarding the space group is based on X-ray diffraction scaling statistics and the cell and point group of the dataset. For example, *XCE* reads the PDB header of each declared polymorph and determines the point group and unit cell volume. Then, once processing each fragment-soaked dataset, if the MTZ point group is identical to the one of a reference polymorph and the unit cell volume is similar (within 12%, but this can be tuned), it assumes that they belong to the same crystal form. *XCE* also allows for the detection of unexpected crystal forms for a subset of the collected crystals: in the presence of different polymorphs, discrepancies between the reference files provided and some of the datasets arise—either in terms of the space groups and/or the unit cells’ volumes or because of high R_Rfree_ values after initial refinement. In the presence of crystal polymorphism, a certain degree of manual curation is, therefore, needed to run *XCE* successfully.

When faced with processing hundreds of datasets belonging to different and yet related crystal forms, we reasoned that from a Bayesian statistical standpoint (Bricogne, 1997), the best way of deciding on the correct crystal polymorph is the one that consults all the available data. In this conceptual framework, the likelihood of a certain dataset belonging to one of the known polymorphs can be best evaluated by refining, in turn, each known polymorphic structure against the dataset processed in that symmetry/cell and then choosing the one with the best refinement R_free_ statistic.

## 3 Results on the *Ct*UGGT_*GT*24_ FBLD effort

More than 1,200 *Ct*UGGT_*GT*24_ crystals were grown, and 768 of them were soaked with as many compounds of the DSi-Poised Library (Diamond-SGC-iNext, ex DSPL, https://www.diamond.ac.uk/Instruments/Mx/Fragment-Screening/Fragment-Libraries/DSi-Poised-Library.html).

Each compound was at a concentration of 500 mM in deuterated DMSO; 40 nl of compound stock solution was dispensed by the Echo robot onto each 200-nl drop, making the final compound concentration 83 mM in 20% DMSO.

Automated data collection was carried out on 692 soaked crystals, but only 493 scaled datasets were obtained, spanning the resolution range of 1.72–11.4 Å. The majority of datasets diffracted to 3.5 Å  resolution or better, see Figure 5A.

Only for 439 of these 493 datasets, a structure could be successfully refined in either H3 or P1 or both. Table 1 reports the overall counts, while Figures 5A,B report a few statistics for the 439 diffraction datasets that refined better either in H3 (390 datasets) or P1 (49 datasets).

On average, H3 crystals diffract better than P1 ones: < ^H3^Resolution > = 2.53 Å  and < ^P1^Resolution > = 3.62 Å (Figure 5A). Indeed, the scaling statistics are on average better in H3 than in P1: < ^H3^R_meas_ > = 0.171 and < ^P1^R_meas_ > = 0.341 (Figure 5B).

None of the 39 P1 crystals revealed any bound fragments. The P1 2.05 Å  structure of a co-crystal of the *CtUGGT*_*GT*24_  domain with the UDP–Glc analogue (and UGGT inhibitor) UDP-2-deoxy-2-fluoro-D-glucose (U2F) was obtained after the FBLD effort and is available at the Protein Databank (https://www.rcsb.org) as PDB ID 7ZLU.

Three of the H3-soaked crystals turned out to contain density for a bound *CtUGGT*_*GT*24_ ligand.

### 3.1 Fragment x0441

**FIGURE 4:**
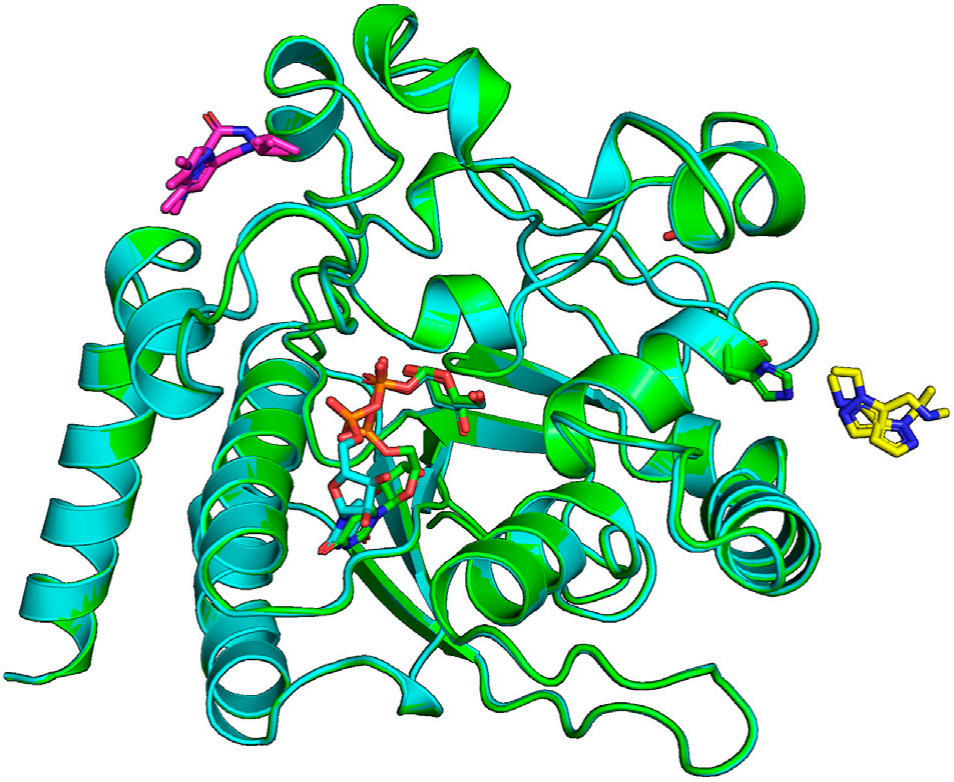
Ligands x0441 and x0763 bind to surface pockets of the *Ct*UGGT_*GT*24_ domain. The crystal structures of *Ct*UGGT_GT24_^UDP−Glc^ soaked in compounds x0441 and x0763 are painted in green and cyan cartoon representation, respectively. The molecule of UDP–Glc in the catalytic pocket of each structure is represented in sticks (its C atoms also in green and cyan for the x0441 and x0763 soaked crystal structure, respectively; N atoms in blue, O atoms in red, and P atoms in orange). Two partially overlapping poses of compound x0673 are represented in sticks with magenta C atoms. Two partially overlapping poses of compound x0441 are represented in sticks with yellow C atoms, next to *Ct*UGGT_*GT*24_ residue H1042, also represented in sticks.

A *Ct*UGGT_GT24_^UDP−Glc^ crystal soaked in (1-(1-ethyl-1H-pyrazol-5-yl)-N-methylmethanamine (SMILES string CCN1N=CC=C1CNC, “fragment x0441”) shows density for the ligand at a crystal contact, but the binding site is not particularly conserved: *Ct*UGGT Y1350, H1402, Q1381, and M1403 (which in *Hs*UGGT1 corresponds to H1406, N1458, Q1437, and M1459) (Figures 1, 4). The pose is rather ambiguous at this resolution (2.44 Å). This site, in the context of the full-length UGGT molecule, faces the UGGT central saddle and is proximal to the putative binding site of the first GlcNAc of the UGGT client’s glycan (close to *Ct*UGGT H1402, which is *Hs*UGGT1 N1508).

### 3.2 Fragment x0763

The *Ct*UGGT_GT24_^UDP−Glc^ crystal soaked in (1,3-dimethyl-N-(propan-2-yl)-1H-pyrazole-5-carboxamide (SMILES string CC(C)NC(=O)C=1C=C(C)N(C)N1, “fragment x0763”) also showed residual electron density for the ligand (in two orientations), this time in a conserved pocket: *Ct*UGGT T1442, M1441, R1333, R1452, and E1444 (which in *Hs*UGGT1 corresponds to T1498, M1497, R1389, R1508, and E1500) (Figures 1, 4). The pocket is not far from the putative binding site for the C-branch of the client glycoprotein glycan (*Ct*UGGT M1336, Y1339, and M1441 or *Hs*UGGT1 M1392, Y1395, and M1507), but the interactions of the fragment with the GT domain are weak—perhaps because the binding site is at a crystal contact.

### 3.3 Fragment x0248: *Ct*UGGT_GT24_^5M−8OH−Q^ crystal structure

The best hit of the FBLD effort was 5-[(morpholin-4-yl)methyl]quinolin-8-ol (SMILES string C1COCCN1CC2=C3C=CC=NC3=C(C=C2)O, “fragment x0248,” henceforth 5M-8OH-Q), a crystal soaked with which diffracted to 2.25 Å  (PDB ID 7ZXW). Diffraction data from this crystal process in H3 with R_meas_^H3^ = 0.323 and in P1 with R_meas_^P1^ = 0.206. The *CCP4-Dimple*/*REFMAC5* R_free_s are R_free_^H3^ = 0.227 and R_free_^P1^ = 0.2537.

The compound binds to a conserved patch on the surface of the *Ct*UGGT_*GT*24_  domain, about 15 Å  away from the UDP–Glc binding site (Figure 3B). The morpholine ring is partially disordered in the crystal, but one of its ring placements is 4.2 Å  from the conserved ^1396^DQD^1398^ motif coordinating the Glc ring of UDP–Glc; the ligand also causes a displacement of the side chain of *Ct*UGGT_*GT*24_  ^1346^Y. Through this displacement, the 8OH-quinoline ring inserts and is sandwiched between the aromatic side chains of the conserved residues ^1346^YW^1347^—which we propose to call the “YW clamp.” The two aromatic side chains stabilise the quinoline ring, forming an aromatic trimer (Lanzarotti et al., 2020); the 8-OH group of the quinoline also establishes an H-bond to the side chain of ^1402^H (Figure 3B).

Tables 2, 3 report the crystallographic data and refinement statistics for the *Ct*UGGT_GT24_^UDP−Glc^ and *Ct*UGGT_GT24_^5M−8OH−Q^ crystal structures.

**TABLE 2:** X-ray data collection parameters and data processing statistics for *Ct*UGGT_*GT*24_ crystal structures.

Structure	*Ct*UGGT_GT24_^UDP−Glc^	*Ct*UGGT_GT24_^5M−8OH−Q^
PDB ID	6FSN	7ZXW
Beamline	I03@DLS	I04-1@DLS
Wavelength *λ* (mm, Å)	0.97960	0.91587
Transmission %	100	100
Number of images	1,800	1,000
Oscillation range (°)	0.1	0.18
Exposure time (s)	0.02	0.06
Space group (Z)	H3 (6)	H3 (6)
Cell edges: a, b, and c (Å)	a = b = 118.83 and c = 68.75	a = b = 118.01,c = 68.37
Cell angles *α*, *β*, *γ* (°)	*α* = *β* = 90, *γ* = 120	*α* = *β* = 90, *γ* = 120
Resolution range (Å)	57.17–1.27 (1.34–1.27)	59.00–2.53 (2.37–2.25)
R_ merge_	0.07 (1.40)	0.29 (1.95)
R_ meas_	0.08 (1.56)	0.32 (2.16)
Observations	486,484 (68,419)	86,970 (12,914)
Unique observations	94,855 (13,700)	16,926 (2,464)
Average I/*σ*(I)	10.5 (1.1)	6.1 (1.2)
Completeness %	99.0 (97.7)	99.9 (99.9)
Multiplicity	5.1 (5.0)	5.1 (5.2)
CC_1/2_	0.99 (0.50)	0.98 (0.37)

Each structure was determined using a single crystal. Values in parentheses refer to the highest resolution shell.

**TABLE 3:** Refinement statistics for *Ct*UGGT_*GT*24_ crystal structures. All structures contain a Ca^+^ ion coming from the protein solution.

Structure	*Ct*UGGT_GT24_^UDP−Glc^	*Ct*UGGT_GT24_^5M−8OH−Q^
PDB ID	6FSN	7ZXW
Wavelength *λ* (Å)	0.97960	0.91587
Space group (Z)	H3 (6)	H3 (6)
Resolution range (Å)	57.17–1.27 (1.34–1.27)	59.01–2.25 (2.26–2.25)
R_work_, R_free_	0.211 (0.227)	0.202 (0.240)
Protein atoms ( < B factor > , Å^2^)	2,426 (25.5)	2,380 (34.5)
Water molecules ( < B factor > , Å^2^)	252 (34.69)	194 (40.5)
Ligands ( < B factor > , Å^2^)	Ca^2+^, UDP-Glc (20.44)	Ca^2+^, UDP-Glc, 5M-8OH-Q (59.2)
rmsd_bonds_ (Å), rmsd_angles_ (^◦^)	0.01, 1.06	0.008, 0.95

Each structure was determined using a single crystal. Values in parentheses refer to the highest resolution shell.

## 4 Discussion

In the course of an X-ray crystallography FBLD effort aimed at the discovery of molecules binding the catalytic domain of *Chaetomium thermophilum* UGGT (*Ct*UGGT_*GT*24_), we encountered crystal polymorphism: the majority of *Ct*UGGT_*GT*24_  crystals belonged to space group H3, but some crystals lowered their symmetry to an equivalent P1 pseudo-rhombohedral lattice. The space group and lattice ambiguity was tricky to resolve by the automated FBLD data processing algorithm—which essentially did not commit to a specific polymorph and left the polymorph choice to the user. However, how is such a choice best implemented in an automated and reliable fashion?

First, if the coordinates of enough indexing spots are gathered, the reciprocal cell parameters can be rather precise. Errors in the reciprocal lattice choice can then be estimated by the differences between reciprocal cell parameters that would differ in the two reciprocal lattices being compared. For example, for the *Ct*UGGT_*GT*24_ crystals described in this study, b* and c* would be identical in R3 but differ in P1.

In cases where alternative lattices are pseudo-equivalent and symmetry/cell changes are such that the volume of the asymmetric unit increases/decreases, a discriminating criterion between polymorphs can be based on indexing quality or average intensity of classes of reflections that are systematically extinct in one polymorph and allowed in another one. For example, if indexing and integration of a lattice-centred dataset are carried out in a primitive lattice, the data will appear pseudo-lattice-centred, with more reflections in the primitive than in the lattice-centred space group, and the question that will help discriminate the space groups is then how strong are the additional reflections only allowed in the primitive cell polymorph compared to those that are present in both polymorphs. The values of the fractional mean intensity of the additional reflections, perhaps as a function of resolution, would help discriminate between a truly centred lattice and a pseudo-centred one.

In the general case in which the polymorphs have equivalent lattices but different symmetries, or unrelated lattices altogether, a polymorph choice based on indexing quality (or average intensity of classes of reflections) may not be so straightforward.

A second class of statistics that may be conceivably used for polymorph discrimination are scaling statistics, but each commonly evaluated scaling statistic risks opening a different can of worms if chosen as the basis for polymorph discrimination. For example, R_merge_ has been shown to privilege lower-symmetry space groups, and R_meas_ was introduced to discriminate between space groups with different scaling multiplicities (Diederichs and Karplus, 1997; Karplus and Diederichs, 2015). Unfortunately, a robust estimation of R_meas_ requires data multiplicity—and so does the estimation of CC_1/2_ (Karplus and Diederichs, 2015): both scaling statistics are strongly dependent on random (*i.e.*, photon-counting) errors and may not discriminate very well between low-signal but correct symmetry and high-signal but wrong symmetry. Data multiplicity will be high enough for reliable polymorph choice (in the presence of polymorphs of low symmetry) only if more than half a reciprocal sphere of data is collected and/or a data collection strategy is followed—both of which are uncommon practices in most FBLD efforts. For example, in P1, two (or more) datasets of 180^◦^ each, with a large kappa offset, would be needed. Data of this kind may not be always available for low-symmetry space group FBLD datasets, thus ruling out the implementation of strategies based on statistics like R_meas_ or CC_1/2_ or those where the error model comes into play, like average *I*/*σ*(*I*) and its sister statistic, asymptotic I/*σ*(I)_asympt_ (ISa) (Diederichs, 2010). Supplementary Figure S1 illustrates scatter plots of outer shell *I*/*σ*(*I*), outer shell CC_1/2_, I/*σ*(I)_asympt_ (ISa), and R_meas_ separately for *Ct*UGGT_*GT*24_  datasets that had lower R_free_ in H3 or P1. None of these data processing-based statistics would lead to polymorph choice in overall agreement with the R_free_ one.

We decided to test a polymorph choice strategy that would consult the known H3 and P1 atomic structures, and for each dataset use both of them individually in refinement against the diffraction data (processed in that symmetry), in order to resolve the H3 vs. P1 decision, which would then have the advantage of being based on all the prior information available (Jaynes, 1968).

Polymorph choice based on refinement statistics of course has its own risks/drawbacks. First, reliable refinements in all possible polymorphs depend on the availability of a structural model of good quality for each of them. Second, when the lattices of the polymorphs at hand are related, to properly compare R_free_ values, the free set of reflections should be chosen consistently: for example, the free set of a lower-symmetry polymorph should be a symmetry expansion of the pseudo-equivalent higher-symmetry one. Finally, the standard error of an R value is (roughly) inversely related to the square root of the number of reflections. So how big does a difference of R_free_ values have to be so that it is statistically significant? A statistical test would be needed to judge if the improvement on R_free_ warrants the choice of the lower-symmetry space group over the higher-symmetry one.

More generally, we are aware that, strictly speaking, the use we make of R_free_ is not what the statistic was invented for (Brunger, 1992), that is, discrimination between alternative models in view of one set of diffraction structure factor amplitudes. In the case of polymorph choice, the single set of data to account for are the diffraction images, while the alternative models to be tested against the diffraction data comprise lattice parameters (including the orientation matrix) and space group symmetry as well as the usual atomic model parameters.

The best approach to the problem would, therefore, require a single integrated piece of software that refines two classes of parameters against unprocessed X-ray diffraction images: the first class of parameters are the ones traditionally refined during X-ray data processing, and the second class are the parameters traditionally refined during macromolecular refinement. To be most useful for the purpose of polymorph choice, such a program would likely need to deal at least with some of the correlations between parameters belonging to either class (Roversi and Tronrud, 2021). Each refinement of the atomic structures of one of the possible polymorphs directly against the diffraction images would compute a R_free_, a free likelihood or another model comparison metric, (Babcock et al., 2018) and presumably enable the choice of the best polymorph as the one that uses only as few parameters as are needed to fit the signal but not the noise in the data, and no more.

**FIGURE 5:**
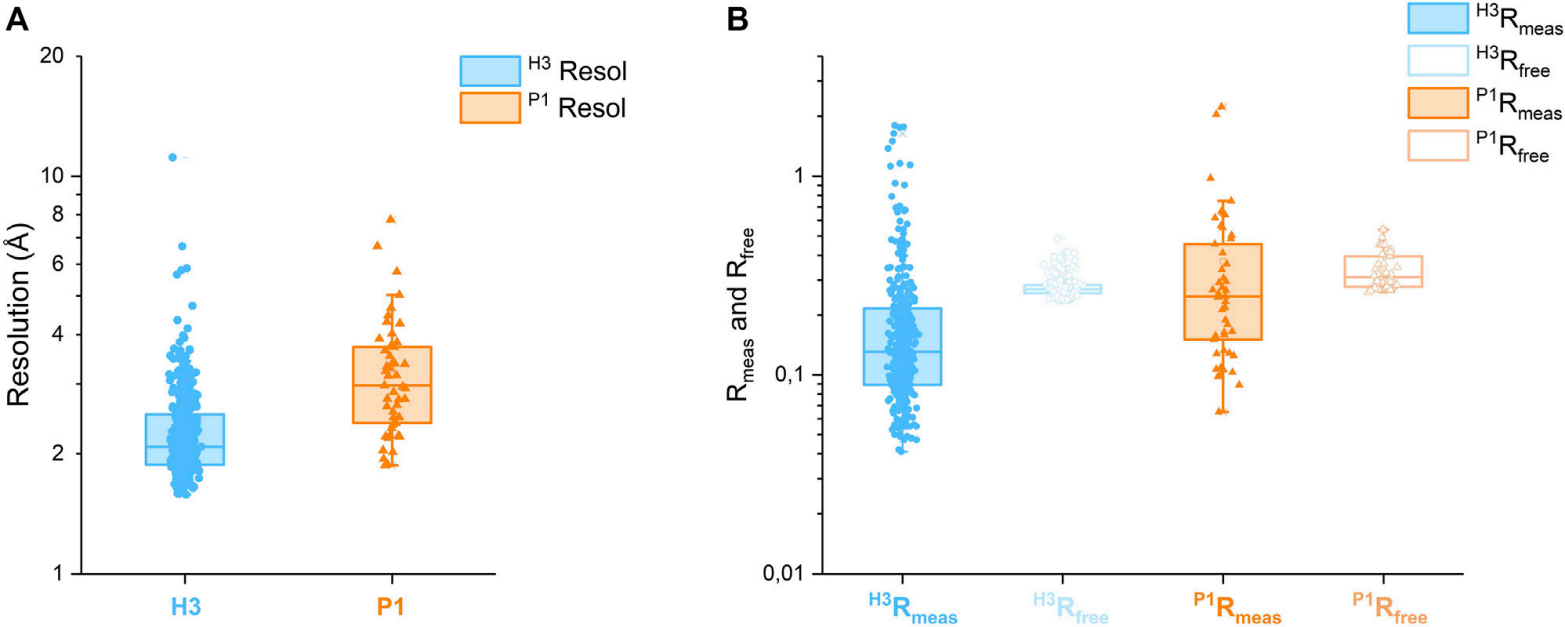
**(A)** Box plots of resolution for 390 *Ct*UGGT_*GT*24_ FBLD datasets, which refined better in H3, and 49 datasets, which refined better in P1. **(B)** Box plots of R_meas_ and R_free_ for the same *Ct*UGGT_*GT*24_ FBLD datasets. Both the resolution and R scales are logarithmic.

Rather than a statistically solid (and likely time-consuming!) solution to the problem of polymorph choice in FBLD, we have aimed here at the implementation and testing of a simple protocol that would nevertheless choose polymorphs by consulting the information in the available atomic models. The scripts implementing the automated polymorph choice processed each of the 493 *Ct*UGGT_*GT*24_ datasets both in H3 and in P1, enabling 439 structural refinements to take place in either or both space groups. For the 156 datasets that were refined in both H3 and P1, the polymorph choice was then based on the symmetry giving the lower R_free_. For each dataset, in each polymorph, our scripts inherit the free set of reflections from the reference dataset in that polymorph so that the choice of free sets for a pair of related-lattice polymorphs can be made consistently once and for all before running the refinements on which the polymorph choice is based. Unsurprisingly, analysis of the distributions of R_meas_ and R_free_ over these datasets reveals that in both polymorphs, the dispersion of the latter statistic is sharper than the one of the former (see Figure 5B), supporting the choice of the polymorph based on R_free_ rather than R_meas_. The best hit was a *Ct*UGGT_*GT*24_  2.2 Å  H3 crystal soaked in 5M-8OH-Q. The molecule is bound to a conserved patch on the surface of the protein. The compound is now the starting point for a medicinal chemistry programme that will develop more potent and selective UGGT inhibitors.

As to the generality/feasibility of our strategy, it is true that its computational requirements scale linearly with the number of possible polymorphs. For a given dataset and n possible polymorphs, all our approach requires is full data integration, scaling, and refinement in each polymorph. Synchrotron high-throughput automated data processing pipelines run such a series of steps many times per dataset already: for example, at some synchrotrons, the *IspyB* system (Monaco et al., 2013) enables data reduction and storage of each dataset at least three times: with *xia2* and *xia2. multiplex* (Winter et al., 2013; Gildea et al., 2022), *DIALS* (Winter et al., 2022), and *autoPROC* (Vonrhein et al., 2011), run with a variety of defaults and making use of programs such as *XDS* (Kabsch, 2010) and/or toolkits such as *CCTBX* (Grosse-Kunstleve et al., 2002). Each of these data processing strands is then completed by a structural refinement. One of the reasons for this “redundancy” is indeed related to the possibility that different data processing algorithms may choose different polymorphs, thus alerting the user to the possibility of alternative symmetries/lattices. As we do not know of examples of fragment-based lead discovery efforts encountering more than *n* = 3 polymorphs, one possible solution to make a strategy equivalent to the one we suggest and yet retain generality and feasibility would be commitment to one of the currently implemented data processing pipelines and its repetition for each of n possible polymorphs. In most cases, if n is not large, such an approach would not add much extra time, and it would still systematically sample all possible polymorphs.

Whichever the best solution, implementation of automated polymorph assignment will be an important step towards the realisation of the full potential of crystal polymorphism in FBLD (Vera et al., 2013). It is our hope that with minimal tweaks, existing pipelines for FBLD data processing can be modified to implement ideas similar to the ones we have described here, when discriminating between polymorphs which are related and, therefore, difficult to distinguish on the basis of cell parameters, diffraction data apparent point group, and scaling statistics alone.

## 5 Materials and methods

### 5.1 *Ct*UGGT_*GT*24_ cloning, protein expression, and purification

The C-terminally 6xHis-tagged *Ct*UGGT_*GT*24_ construct corresponding to *Ct*UGGT residues 1,187–1,473 was successfully amplified by PCR starting from the *Ct*UGGT–pHLSec vector (Roversi et al., 2017) using primers *Ct*UGGT_*GT*24__Fwd: ggt​tgc​gta​gct​gaa​acc​ggt​GAG​GCA​ACC​AAG​TCC​GTG and *Ct*UGGT_*GT*24__Rev: gat​ggt​ggt​gct​tgg​tac​cTT​CCC​TCA​CTC​TCC​TCG​C.

The amplified insert was identified by agarose gel electrophoresis (about 900 bp) and purified from the gel.

Following purification of the PCR products, the *CtUGGT*_*GT*24_  insert was assembled into the AgeI/KpnI linearised pHLSec vector (Aricescu et al., 2006) *via* ligation-independent cloning (*aka* Gibson assembly). After transformation and plating, *E. coli* colonies containing the desired construct were identified by colony PCR through identification by agarose gel electrophoresis of the correct size. *Ct*UGGT–pHLsec DNA plasmid purification from the correctly identified colonies was carried out *via* DNA miniprep and the resulting plasmid DNA sent for sequencing for confirmation of the desired DNA construct.

The maxiprepped *Ct*UGGT–pHLsec DNA plasmid was transfected into HEK293F cells following the protocol used for *Ct*UGGT (Roversi et al., 2017). Purification was achieved by IMAC on an Åkta FPLC system, followed by gel filtration chromatography, after which the proteins were identified by SDS-PAGE. The final buffer was 20 mM HEPES pH 7.4, 50 mM NaCl, and 1 mM CaCl_2_.

### 5.2 *Ct*UGGT_*GT*24_ co-crystallization with UDP-Glc

All crystals were grown at 18°C in sitting drops by the vapour diffusion method, set up with a mosquito liquid handling robot (TTP Labtech). Crystallisation drops had an initial volume of 200 nl. The volume ratio of protein to precipitant was either 1:1 or 2:1.

Crystals of *Ct*UGGT_GT24_^UDP−Glc^ grew in 1 week in a 1:1 mixture of *Ct*UGGT at 6 mg/ml, 2 mM CaCl_2_, and 5 mM UDP–Glc and a number of Morpheus screen conditions (Gorrec, 2009, Gorrec, 2015).

The best crystals came from a crystal grown in Morpheus screen condition 2–9 containing 0.12 M ethylene glycol, 0.1 M buffer system 3 pH 8.5, and 30% v/v precipitant mix 1 (Gorrec, 2009; Gorrec, 2015).

### 5.3 *Ct*UGGT_GT24_^UDP−Glc^  crystal growth for FBLD

A 96-well deep well block was prepared with 500 *μ*l in each well: 257.5 *μ*l of Morpheus precipitant mix 1 (40% v/v PEG 500 MME; 20% w/v PEG 20000); 25.8 *μ*l of 1 M Bicine (buffer system 3 acid component); 24.3 *μ*l of 1 M Tris (buffer system 3 basic component); 192.4 *μ*l of MilliQ Water. The Hydra robot at the Research Complex in Harwell was first used to transfer 25 *μ*l of the crystallisation solution from the deep well block to each mother liquor well of six MRC 3-well crystallisation plates. Vapour diffusion experiments were set up at the Research Complex in Harwell in six MRC 3-well crystallisation plates—using a mosquito robot equipped with an anti-evaporation cover with 60% controlled humidity in order to avoid drying up of the crystallisation drops during deposition. A total of 6 × 96 × 3 = 1728 drops were set up. The *Ct*UGGT_*GT*24_ protein at a concentration of 6.5 mg/ml, in the presence of 1 mM CaCl_2_ and 5 mM UDP–Glc), was mixed in protein: mother liquor proportions 1.35:1 (drops a and c) and 2:1 (drop d) in drops of total volume 200 nl, and the crystals were left to grow at 18°C. In less than a week, about two-thirds of the experiments yielded crystals.

### 5.4 *Ct*UGGT_GT24_^UDP−Glc^ crystal soaking for FBLD

Prior to the fragment-based lead discovery effort, 50 of the *Ct*UGGT_GT24_^UDP−Glc^ crystals were soaked in 0, 5, 10, 15, 20, and 30% DMSO and diffraction tested; no significant deterioration of the diffraction quality was observed. The space group prior to soaking is H3, one molecule per asymmetric unit, with cell edges a = b = 119 Å, and c = 69 Å.

All *Ct*UGGT_GT24_^UDP−Glc^ crystal drops were imaged, and the best crystals were marked for soaking with the Echo robot at the Xchem facility attached to beamline I04-1 at the Diamond Light Source (Collins et al., 2017; Douangamath et al., 2021). 768 compounds of the DSi-Poised Library (Diamond-SGC-iNext, ex DSPL https://www.diamond.ac.uk/Instruments/Mx/Fragment-Screening/Fragment-Libraries/DSi-Poised-Library.html) and compound stock solutions at a concentration of 500 mM in deuterated DMSO were each soaked into a crystal drop: 40 nl of the compound was dispensed by the Echo robot (Collins et al., 2017) onto each 200-nl drop, making the final compound concentration 83 mM in 20% DMSO. The soaked crystals were left incubating for a variable time between 2 and 4 h. 692 crystals were fished and cryocooled with the aid of the SGC Shifter Robot (Wright et al., 2021).

### 5.5 *Ct*UGGT_GT24_^UDP−Glc^ soaked crystal X-ray diffraction for FBLD

Automated data collection was carried out on 596 soaked crystals. Automated loop centring failed about 6% of the time, and about 50 crystals were re-measured with optical centring. The symmetry is sometimes lowered by soaking, the crystal can in this case index in space group P1 with three molecules in the asymmetric unit of a pseudo-rhombohedral cell of dimensions a = 66.3 Å b = 72.7 Å c = 72.7 Å, alpha = 111.2°, beta = 107.7°, and gamma = 107.7°. This cell is related to the one of the rhombohedral setting of the H3 crystals, a = b = c = 72.32 Å  and *α* = *β* = *γ* = 110.43°.

### 5.6 *Ct*UGGT_GT24_^UDP−Glc^ X-ray data processing, model refinement, and ligand fitting for FBLD

Data processing, model refinement, and ligand fitting were carried out with the purpose-written shell script pipeline *CoALLA*. In order to decide on the correct symmetry, each dataset was indexed and integrated both in H3 and in P1 using the *autoPROC* suite of programmes; for each dataset, refinement of the protein model was carried out in both the H3 and P1 form in *autoBUSTER*, with the space group giving rise to the lower Rfree being chosen as the correct one for the calculation of the Fo–Fc map. The SMILES string for each compound was fed together with the refined model and phases in order to attempt docking the ligand using *rhofit*. The best hits were listed by ranking the *rhofit* score and/or CC and the hits inspected in *Coot*.

#### 5.6.1 Data processing and polymorph assignment

In order to run *CoALLA*, all possible polymorphs must be known; moreover, two reference files must be available for each polymorph: one X-ray diffraction dataset with experimental structure factor amplitudes [in mtz format (Winn et al., 2011)] and its corresponding structure coordinates (in PDB format).

The *CoALLA* pipeline initially processes the diffraction data in each and every one of the polymorphs listed in input, using the data processing suite *autoPROC* Vonrhein et al. (2011).

The resolution of the data processed in each polymorph is initially chosen by *autoPROC* using the *autoPROC* command flag -M HighResCutOnCChalf, which by default sets the maximum resolution so that CC_1/2_ in the outer shell is no lower than 0.30.

At the indexing stage, in order to ensure consistent indexing across all FBLD datasets belonging to the same polymorph, the *autoPROC* command line flag -ref <reference dataset>—is used, enforcing the same indexing choice as the reference diffraction dataset for the polymorph being tested. If the reference cell dimensions differ significantly from any of the autoindexed ones, *autoPROC* may be unable to refine a reference-based indexing solution that fits the data: the reference dataset indexing choice is then enforced only after *autoPROC* indexing/integration by a run of *CCP4-pointless* (Evans, 2011) with the keyword TOLERANCE 10^1^. At this stage, the reference dataset R_free_ flags are also inherited for each polymorph (using *CCP4-CAD*) and are kept for all subsequent calculations for the dataset in that polymorph.

**FIGURE 6:**
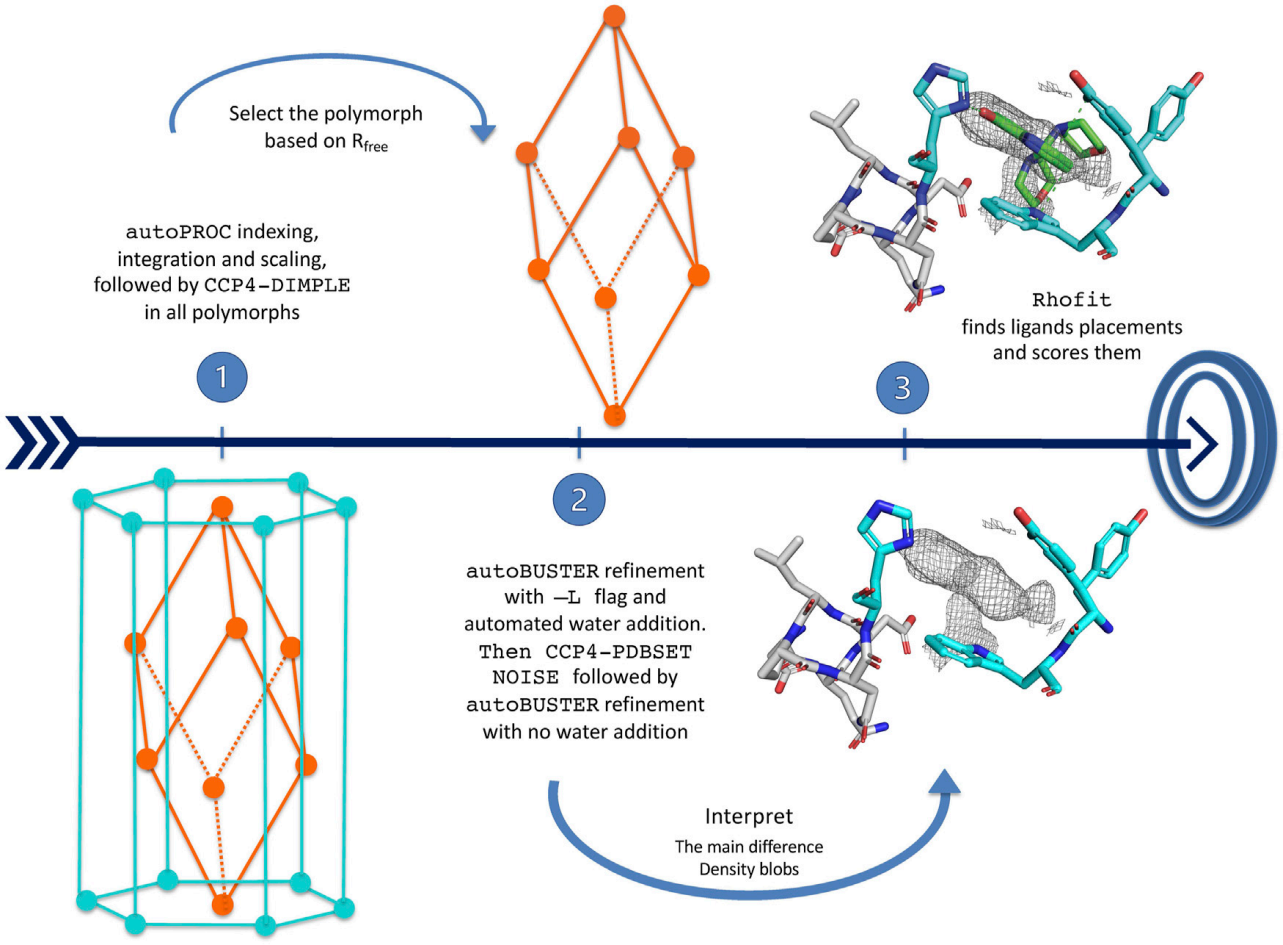
*CoALLA* flow diagram.

The *PanDDA* pipeline (Pearce et al., 2017a; Pearce et al., 2017b; Pearce et al., 2017c) in use at Diamond Light Source beamline I04-1 Douangamath et al. (2021)—where the data were collected—uses *CCP4-Dimple*/*REFMAC5* (Murshudov et al., 2011; Winn et al., 2011; Keegan et al., 2015) as its refinement engine (see Figure 6). At the initial stage of deciding on the correct polymorph, it seemed natural to exploit the fast refinement capabilities of *CCP4-Dimple*. After data processing in all polymorphs, the hypothesis that the data belong to a certain polymorph is tested in *CoALLA* by running structural refinement in *CCP4-Dimple* (Winn et al., 2011; Keegan et al., 2015) against the data processed in each polymorph in turn.

In each polymorph, the *CoALLA* scripts run two *CCP4-Dimple* refinements of the reference model against the dataset as processed in that polymorph: a rigid body refinement first, followed by a full atomic one. In order to be able to compare the R_free_s of models refined in different polymorphs, all *CCP4-Dimple* refinements are run in *CoALLA* up to the best common maximum resolution limit across all polymorphs, which the scripts obtain for each polymorph by harvesting the resolution limit from the relevant *autoPROC* output file (see Figure 6).

Once a *CCP4-Dimple* refinement of the structure has run for each polymorph, the polymorph with the best *CCP4-Dimple* refinement R_free_ is chosen as the most likely one to continue the analysis. It is at this stage that *CoALLA* improves on automated polymorph choices based on data processing only: the available information about the structures of the polymorphs is added to the one present in the diffraction data to single out the most likely polymorph.

#### 5.6.2 *BUSTER* structure refinements

The structures are then refined in *BUSTER* starting from the *CCP4-Dimple* output file, with automated NCS restraints and external restraints to the reference structure (Smart et al., 2012), command line keywords—target <ref.pdb>—autoncs).

The first *BUSTER* refinement is run with the -L command line flag: this turns on automated water updating. Waters initially also fill volumes where potential ligands are located, but the water atoms around residual difference density are then removed at the last *BUSTER* refinement cycle. This procedure enables water-building without deteriorating the quality of the difference density in putative ligand regions.

At this stage, *CCP4-PDBSET* is run on the *BUSTER*-refined model (with keyword NOISE) in order to introduce positional noise in the coordinates resulting from the refinement previously described—including any waters that survived the pruning at the end of the previous *BUSTER* refinement with automated water addition. The positional noise introduced by *CCP4-PDBSET*at this stage ought to wipe out any “memory” of the waters that may have been refined (and then were removed) in putative ligand pockets. A second *BUSTER* refinement (without water addition) is then run starting from the *CCP4-PDBSET* shaken model. The 2Fo–Fc and Fo–Fc density maps necessary for ligand identification and docking are computed at the end of this *BUSTER*  refinement.

#### 5.6.3 *RHOFIT* ligand placement

Automatic interpretation of crystal residual difference density fully automates the placement of ligands, providing an unbiased alternative to manual ligand placement, especially for data of low to medium resolution (Langer et al., 2008; Carolan and Lamzin, 2014; Echols et al., 2014; Wlodek et al., 2006). Algorithms can take into account protein–ligand interactions as well as fit to the map (Mooij et al., 2006).

The *RHOFIT* program (Smart et al., 2014) is used within the *CoALLA* pipeline to find the best placements in all the *BUSTER*  refined maps.

## Data Availability

The datasets presented in this study can be found in online repositories. The names of the repository/repositories and accession number(s) can be found at: http://www.wwpdb.org/, 6fsn http://www.wwpdb.org/, 7zxw.

## Footnotes

1 Note that in *Phenix*-based FBLD pipelines, models are mapped to the same frame of reference as an isomorphous structure. The *CoALLA* algorithm choice of enforcing indexing to the polymorph’s reference dataset has the advantage that simple rigid-body refinement of the reference structure against the data processed in the chosen polymorph is enough to commit to consistent origin choices for all crystals belonging to a certain polymorph, without the need for any molecular replacement steps and/or origin reconciliation.

## Supplementary material

The Supplementary Material for this article can be found online at: https://www.frontiersin.org/articles/10.3389/fmolb.2022.960248/full#supplementary-material. The CoALLA shell script will be available from the corresponding authors upon request.

## References

[B1] Albesa-Jové D. Guerin M. E. (2016). The conformational plasticity of glycosyltransferases. Curr. Opin. Struct. Biol. 40, 23–32. Carbohydrate–protein interactions and glycosylation • Biophysical and molecular biological methods. 10.1016/j.sbi.2016.07.007 27450114

[B2] Alonzi D. S. Scott K. A. Dwek R. A. Zitzmann N. (2017). Iminosugar antivirals: The therapeutic sweet spot. Biochem. Soc. Trans. 45, 571–582. 10.1042/BST20160182 28408497 PMC5390498

[B3] Amara J. F. Cheng S. H. Smith A. E. (1992). Intracellular protein trafficking defects in human disease. Trends Cell. Biol. 2, 145–149. 10.1016/0962-8924(92)90101-r 14731969

[B4] Aricescu A. R. Lu W. Jones E. Y. (2006). A time- and cost-efficient system for high-level protein production in mammalian cells. Acta Crystallogr. D. Biol. Crystallogr. 62, 1243–1250. 10.1107/S0907444906029799 17001101

[B5] Babcock N. S. Keedy D. A. Fraser J. S. Sivak D. A. (2018). Model selection for biological crystallography. bioRxiv. 10.1101/448795

[B6] Blanc E. Roversi P. Vonrhein C. Flensburg C. Lea S. M. Bricogne G. (2004). Refinement of severely incomplete structures with maximum likelihood in BUSTER-TNT. Acta Crystallogr. D. Biol. Crystallogr. 60, 2210–2221. 10.1107/S0907444904016427 15572774

[B7] Bricogne G. Blanc E. M. B. Flensburg C. Keller P. Paciorek W. (2017). Transition metals in catalysis: The functional relationship. *BUSTER 2.10.3* .

[B8] Bricogne G. (1997). “The bayesian statistical viewpoint on structure determination: Basic concepts and examples,” in Macromolecular crystallography. Editors Carter C. W. Jr. Sweet R. M. (San Diego, CA: Academic Press), 361–423. vol. 276 of *Methods in Enzymology* . 10.1016/S0076-6879(97)76069-5 27799106

[B9] Brunger A. T. (1992). Free R value: A novel statistical quantity for assessing the accuracy of crystal structures. Nature 355, 472–475. 10.1038/355472a0 18481394

[B10] Buerger M. J. (1936a). The general rôle of composition in polymorphism. Proc. Natl. Acad. Sci. U. S. A. 22, 685–689. 10.1073/pnas.22.12.685 16577752 PMC1076844

[B11] Buerger M. J. (1936b). The kinetic basis of crystal polymorphism. Proc. Natl. Acad. Sci. U. S. A. 22, 682–685. 10.1073/pnas.22.12.682 16577751 PMC1076843

[B12] Caputo A. T. Alonzi D. S. Marti L. Reca I.-B. Kiappes J. L. Struwe W. B. (2016). Structures of mammalian ER *α*-glucosidase II capture the binding modes of broad-spectrum iminosugar antivirals. Proc. Natl. Acad. Sci. U. S. A. 113, E4630–E4638. 10.1073/pnas.1604463113 27462106 PMC4987793

[B13] Carolan C. G. Lamzin V. S. (2014). Automated identification of crystallographic ligands using sparse-density representations. Acta Crystallogr. D. Biol. Crystallogr. 70, 1844–1853. 10.1107/S1399004714008578 25004962 PMC4089483

[B14] Carter C. W. Doublié S. Coleman D. E. (1994). Quantitative analysis of crystal growth. Tryptophanyl-tRNA synthetase crystal polymorphism and its relationship to catalysis. J. Mol. Biol. 238, 346–365. 10.1006/jmbi.1994.1297 8176729

[B15] Chen X. Qin S. Chen S. Li J. Li L. Wang Z. (2015). A ligand-observed mass spectrometry approach integrated into the fragment based lead discovery pipeline. Sci. Rep. 5, 8361. 10.1038/srep08361 25666181 PMC4322365

[B16] Ciulli A. Williams G. Smith A. G. Blundell T. L. Abell C. (2006). Probing hot spots at protein-ligand binding sites: A fragment-based approach using biophysical methods. J. Med. Chem. 49, 4992–5000. 10.1021/jm060490r 16884311

[B17] Collins P. M. Douangamath A. Talon R. Dias A. Brandao-Neto J. Krojer T. (2018). “Chapter eleven - achieving a good crystal system for crystallographic x-ray fragment screening,” in Modern approaches in drug discovery. Editor Lesburg C. A. (Academic Press), 251–264. vol. 610 of *Methods in Enzymology* . 10.1016/bs.mie.2018.09.027 30390801

[B18] Collins P. M. Ng J. T. Talon R. Nekrosiute K. Krojer T. Douangamath A. (2017). Gentle, fast and effective crystal soaking by acoustic dispensing. Acta Crystallogr. D. Struct. Biol. 73, 246–255. 10.1107/S205979831700331X 28291760 PMC5349437

[B19] Cornaciu I. Bourgeas R. Hoffmann G. Dupeux F. Humm A.-S. Mariaule V. (2021). The automated crystallography pipelines at the EMBL HTX facility in Grenoble. J. Vis. Exp. (172), e62491. 10.3791/62491 34152315

[B20] D’Alessio C. Caramelo J. J. Parodi A. J. (2010). UDP-GlC:glycoprotein glucosyltransferase-glucosidase II, the ying-yang of the ER quality control. Semin. Cell. Dev. Biol. 21, 491–499. 10.1016/j.semcdb.2009.12.014 20045480 PMC2883647

[B21] Dalziel M. Crispin M. Scanlan C. N. Zitzmann N. Dwek R. A. (2014). Emerging principles for the therapeutic exploitation of glycosylation. Science 343, 1235681. 10.1126/science.1235681 24385630

[B22] Diederichs K. Karplus P. A. (1997). Improved R-factors for diffraction data analysis in macromolecular crystallography. Nat. Struct. Biol. 4, 269–275. 10.1038/nsb0497-269 9095194

[B23] Diederichs K. (2010). Quantifying instrument errors in macromolecular X-ray data sets. Acta Crystallogr. D. Biol. Crystallogr. 66, 733–740. 10.1107/S0907444910014836 20516626

[B24] Douangamath A. Powell A. Fearon D. Collins P. M. Talon R. Krojer T. (2021). Achieving efficient fragment screening at XChem facility at Diamond light Source. J. Vis. Exp. (171), e62414 10.3791/62414 34125095

[B25] Dwek R. A. Bell J. I. Feldmann M. Zitzmann N. (2022). Host-targeting oral antiviral drugs to prevent pandemics. Lancet 399, 1381–1382. 10.1016/S0140-6736(22)00454-8 35344736 PMC8956295

[B26] Echols N. Moriarty N. W. Klei H. E. Afonine P. V. Bunkóczi G. Headd J. J. (2014). Automating crystallographic structure solution and refinement of protein–ligand complexes. Acta Crystallogr. D. Biol. Crystallogr. 70, 144–154. 10.1107/S139900471302748X 24419387 PMC3919266

[B27] Evans P. R. (2011). An introduction to data reduction: Space-group determination, scaling and intensity statistics. Acta Crystallogr. D. Biol. Crystallogr. 67, 282–292. 10.1107/S090744491003982X 21460446 PMC3069743

[B28] Gildea R. J. Beilsten-Edmands J. Axford D. Horrell S. Aller P. Sandy J. (2022). *xia*2.*multiplex*: a multi-crystal data-analysis pipeline. Acta Crystallogr. D. Struct. Biol. 78, 752–769. 10.1107/S2059798322004399 35647922 PMC9159281

[B29] Gorrec F. (2015). The MORPHEUS II protein crystallization screen. Acta Crystallogr. F. Struct. Biol. Commun. 71, 831–837. 10.1107/S2053230X1500967X 26144227 PMC4498703

[B30] Gorrec F. (2009). The MORPHEUS protein crystallization screen. J. Appl. Crystallogr. 42, 1035–1042. 10.1107/S0021889809042022 22477774 PMC3246824

[B31] Grosse-Kunstleve R. W. Sauter N. K. Moriarty N. W. Adams P. D. (2002). The *computational crystallography toolbox*: Crystallographic algorithms in a reusable software framework. J. Appl. Crystallogr. 35, 126–136. 10.1107/S0021889801017824

[B32] Hammond C. Braakman I. Helenius A. (1994). Role of N-linked oligosaccharide recognition, glucose trimming, and calnexin in glycoprotein folding and quality control. Proc. Natl. Acad. Sci. U. S. A. 91, 913–917. 10.1073/pnas.91.3.913 8302866 PMC521423

[B33] Jaynes E. T. (1968). Prior probabilities. IEEE Trans. Syst. Sci. Cyber. 4, 227–241. 10.1109/TSSC.1968.300117

[B34] Jurnak F. (1985). Induction of elongation factor Tu-GDP crystal polymorphism by polyethylene glycol contaminants. J. Mol. Biol. 185, 215–217. 10.1016/0022-2836(85)90194-9 3900422

[B35] Kabsch W. (2010). “XDS,” in Acta crystallographica. Section D, Biological crystallography, 66 (Chester, England: International Union of Crystallography), 125–132. 10.1107/S0907444909047337 20124692 PMC2815665

[B36] Kaminski J. W. Vera L. Stegmann D. P. Vering J. Eris D. Smith K. M. L. (2022). Fast fragment- and compound-screening pipeline at the Swiss Light Source. Acta Crystallogr. D. Struct. Biol. 78, 328–336. 10.1107/S2059798322000705 35234147 PMC8900825

[B37] Karade S. S. Hill M. L. Kiappes J. L. Manne R. Aakula B. Zitzmann N. (2021). N-substituted valiolamine derivatives as potent inhibitors of endoplasmic reticulum *α*-glucosidases I and II with antiviral activity. J. Med. Chem. 64, 18010–18024. 10.1021/acs.jmedchem.1c01377 34870992

[B38] Karplus P. A. Diederichs K. (2015). Assessing and maximizing data quality in macromolecular crystallography. Curr. Opin. Struct. Biol. 34, 60–68. Carbohydrate-protein interactions • Biophysical and molecular biological methods. 10.1016/j.sbi.2015.07.003 26209821 PMC4684713

[B39] Keegan R. Wojdyr M. Winter G. Ashton A. (2015). *Dimple*: A difference map pipeline for the rapid screening of crystals on the beamline. Acta Crystallogr. A Found. Adv. 71, s18. 10.1107/S2053273315099702

[B40] Krojer T. Talon R. Pearce N. Collins P. Douangamath A. Brandao-Neto J. (2017). The XChemExplorer graphical workflow tool for routine or large-scale protein-ligand structure determination. Acta Crystallogr. D. Struct. Biol. 73, 267–278. 10.1107/S2059798316020234 28291762 PMC5349439

[B41] Langer G. Cohen S. X. Lamzin V. S. Perrakis A. (2008). Automated macromolecular model building for X-ray crystallography using ARP/wARP version 7. Nat. Protoc. 3, 1171–1179. 10.1038/nprot.2008.91 18600222 PMC2582149

[B42] Lanzarotti E. Defelipe L. A. Marti M. A. Turjanski A. G. (2020). Aromatic clusters in protein-protein and protein-drug complexes. J. Cheminform. 12, 30. 10.1186/s13321-020-00437-4 33431014 PMC7206889

[B43] Lima G. M. A. Jagudin E. Talibov V. O. Benz L. S. Marullo C. Barthel T. (2021). *FragMAXapp*: Crystallographic fragment-screening data-analysis and project-management system. Acta Crystallogr. D. Struct. Biol. 77, 799–808. 10.1107/S2059798321003818 34076593 PMC8171072

[B44] Monaco S. Gordon E. Bowler M. W. Delagenière S. Guijarro M. Spruce D. (2013). Automatic processing of macromolecular crystallography X-ray diffraction data at the ESRF. J. Appl. Crystallogr. 46, 804–810. 10.1107/S0021889813006195 23682196 PMC3654316

[B45] Mooij W. Hartshorn M. Tickle I. Sharff A. Verdonk M. Jhoti H. (2006). Automated protein–ligand crystallography for structure-based drug design. ChemMedChem 1, 827–838. 10.1002/cmdc.200600074 16902937

[B46] Müller J. Klein R. Tarkhanova O. Gryniukova A. Borysko P. Merkl S. (2022). Magnet for the needle in haystack: “crystal structure first” fragment hits unlock active chemical matter using targeted exploration of vast chemical spaces. J. Med. Chem. Epub ahead of print. 10.1021/acs.jmedchem.2c00813 36069712

[B47] Murray C. W. Blundell T. L. (2010). Structural biology in fragment-based drug design. Curr. Opin. Struct. Biol. 20, 497–507. 10.1016/j.sbi.2010.04.003 20471246

[B48] Murshudov G. N. Skubák P. Lebedev A. A. Pannu N. S. Steiner R. A. Nicholls R. A. (2011). REFMAC5 for the refinement of macromolecular crystal structures. Acta Crystallogr. D. Biol. Crystallogr. 67, 355–367. 10.1107/S0907444911001314 21460454 PMC3069751

[B49] Oksenych V. Kainov D. E. (2022). Broad-spectrum antivirals and antiviral drug combinations. Viruses 14, 301. 10.3390/v14020301 35215894 PMC8876582

[B50] Pardi N. Weissman D. (2020). Development of vaccines and antivirals for combating viral pandemics. Nat. Biomed. Eng. 4, 1128–1133. 10.1038/s41551-020-00658-w 33293724 PMC8336060

[B51] Pearce N. M. Bradley A. R. Krojer T. Marsden B. D. Deane C. M. von Delft F. (2017a). Partial-occupancy binders identified by the Pan-Dataset Density Analysis method offer new chemical opportunities and reveal cryptic binding sites. Struct. Dyn. 4, 032104. 10.1063/1.4974176 28345007 PMC5336473

[B52] Pearce N. M. Krojer T. Bradley A. R. Collins P. Nowak R. P. Talon R. (2017b). A multi-crystal method for extracting obscured crystallographic states from conventionally uninterpretable electron density. Nat. Commun. 8, 15123. 10.1038/ncomms15123 28436492 PMC5413968

[B53] Pearce N. M. Krojer T. von Delft F. (2017c). Proper modelling of ligand binding requires an ensemble of bound and unbound states. Acta Crystallogr. D. Struct. Biol. 73, 256–266. 10.1107/S2059798317003412 28291761 PMC5349438

[B54] Pearce N. M. Skyner R. Krojer T. (2022). Experiences from developing software for large X-ray crystallography-driven protein-ligand studies. Front. Mol. Biosci. 9, 861491. 10.3389/fmolb.2022.861491 35480897 PMC9035521

[B55] Radoux C. J. Olsson T. S. G. Pitt W. R. Groom C. R. Blundell T. L. (2016). Identifying interactions that determine fragment binding at protein hotspots. J. Med. Chem. 59, 4314–4325. 10.1021/acs.jmedchem.5b01980 27043011

[B56] Roversi P. Marti L. Caputo A. T. Alonzi D. S. Hill J. C. Dent K. C. (2017). Interdomain conformational flexibility underpins the activity of UGGT, the eukaryotic glycoprotein secretion checkpoint. Proc. Natl. Acad. Sci. U. S. A. 114, 8544–8549. 10.1073/pnas.1703682114 28739903 PMC5559018

[B57] Roversi P. Tronrud D. E. (2021). Ten things I `hate' about refinement. Acta Crystallogr. D. Struct. Biol. 77, 1497–1515. 10.1107/S2059798321011700 34866607 PMC8647177

[B58] Sayce A. C. Alonzi D. S. Killingbeck S. S. Tyrrell B. E. Hill M. L. Caputo A. T. (2016). Iminosugars inhibit dengue virus production via inhibition of ER alpha-glucosidases-not glycolipid processing enzymes. PLoS Negl. Trop. Dis. 10, e0004524. 10.1371/journal.pntd.0004524 26974655 PMC4790851

[B59] Schiebel J. Krimmer S. G. Röwer K. Knörlein A. Wang X. Park A. Y. (2016). High-throughput crystallography: Reliable and efficient identification of fragment hits. Structure 24, 1398–1409. 10.1016/j.str.2016.06.010 27452405

[B60] Smart O. S. Womack T. O. Flensburg C. Keller P. Paciorek W. Sharff A. (2012). Exploiting structure similarity in refinement: Automated NCS and target-structure restraints in BUSTER. Acta Crystallogr. D. Biol. Crystallogr. 68, 368–380. 10.1107/S0907444911056058 22505257 PMC3322596

[B61] Smart O. Womack T. Sharff A. Flensburg C. Keller P. Paciorek W. (2014). RHOFIT, version 1.2.4. Cambridge, United Kingdom: Global Phasing Ltd.

[B62] Tax G. Lia A. Santino A. Roversi P. (2019). Modulation of erqc and erad: A broad-spectrum spanner in the works of cancer cells? J. Oncol. 2019, 8384913. 10.1155/2019/8384913 31662755 PMC6791201

[B63] Trombetta E. S. Helenius A. (1999). Glycoprotein reglucosylation and nucleotide sugar utilization in the secretory pathway: Identification of a nucleoside diphosphatase in the endoplasmic reticulum. EMBO J. 18, 3282–3292. 10.1093/emboj/18.12.3282 10369669 PMC1171409

[B64] Trombetta S. E. Bosch M. Parodi A. J. (1989). Glucosylation of glycoproteins by mammalian, plant, fungal, and trypanosomatid protozoa microsomal membranes. Biochemistry 28, 8108–8116. 10.1021/bi00446a022 2532539

[B65] Tyrrell B. E. Sayce A. C. Warfield K. L. Miller J. L. Zitzmann N. (2017). Iminosugars: Promising therapeutics for influenza infection. Crit. Rev. Microbiol. 43, 521–545. 10.1080/1040841X.2016.1242868 27931136 PMC5470110

[B66] Vera L. Antoni C. Devel L. Czarny B. Cassar-Lajeunesse E. Rossello A. (2013). Screening using polymorphs for the crystallization of protein–ligand complexes. Cryst. Growth & Des. 13, 1878–1888. 10.1021/cg301537n

[B67] Vonrhein C. Flensburg C. Keller P. Sharff A. Smart O. Paciorek W. (2011). Data processing and analysis with the autoPROC toolbox. Acta Crystallogr. D. Biol. Crystallogr. 67, 293–302. 10.1107/S0907444911007773 21460447 PMC3069744

[B68] Warfield K. L. Alonzi D. S. Hill J. C. Caputo A. T. Roversi P. Kiappes J. L. (2020). Targeting endoplasmic reticulum *α*-glucosidase I with a single-dose iminosugar treatment protects against lethal influenza and dengue virus infections. J. Med. Chem. 63, 4205–4214. 10.1021/acs.jmedchem.0c00067 32227946

[B69] Winn M. D. Ballard C. C. Cowtan K. D. Dodson E. J. Emsley P. Evans P. R. (2011). Overview of the CCP4 suite and current developments. Acta Crystallogr. D. Biol. Crystallogr. 67, 235–242. 10.1107/S0907444910045749 21460441 PMC3069738

[B70] Winter G. Beilsten-Edmands J. Devenish N. Gerstel M. Gildea R. J. McDonagh D. (2022). DIALS as a toolkit. Protein Sci. 31, 232–250. 10.1002/pro.4224 34747533 PMC8740827

[B71] Winter G. Lobley C. M. C. Prince S. M. (2013). Decision making in xia2. Acta Crystallogr. D. Biol. Crystallogr. 69, 1260–1273. 10.1107/S0907444913015308 23793152 PMC3689529

[B72] Wlodek S. Skillman A. G. Nicholls A. (2006). Automated ligand placement and refinement with a combined force field and shape potential. Acta Crystallogr. D. Biol. Crystallogr. 62, 741–749. 10.1107/S0907444906016076 16790930

[B73] Wright N. D. Collins P. Koekemoer L. Krojer T. Talon R. Nelson E. (2021). The low-cost Shifter microscope stage transforms the speed and robustness of protein crystal harvesting. Acta Crystallogr. D. Struct. Biol. 77, 62–74. 10.1107/S2059798320014114 33404526 PMC7787106

[B74] Yekwa E. Khourieh J. Canard B. Papageorgiou N. Ferron F. (2017). Activity inhibition and crystal polymorphism induced by active-site metal swapping. Acta Crystallogr. D. Struct. Biol. 73, 641–649. 10.1107/S205979831700866X 28777079

[B75] Zabara A. Amar-Yuli I. Mezzenga R. (2011). Tuning in-meso-crystallized lysozyme polymorphism by lyotropic liquid crystal symmetry. Langmuir 27, 6418–6425. 10.1021/la200710p 21506575

