# Supplementary material for: Crystal polymorphism in fragment-based lead discovery of ligands of the catalytic domain of UGGT, the glycoprotein folding quality control checkpoint
Source: Front Mol Biosci. 2022 Dec 14;9:960248. doi: 10.3389/fmolb.2022.960248 (PMC9794592; doi:10.3389/fmolb.2022.960248)
Supplement: Supplementary file 1 [file DataSheet1.PDF]

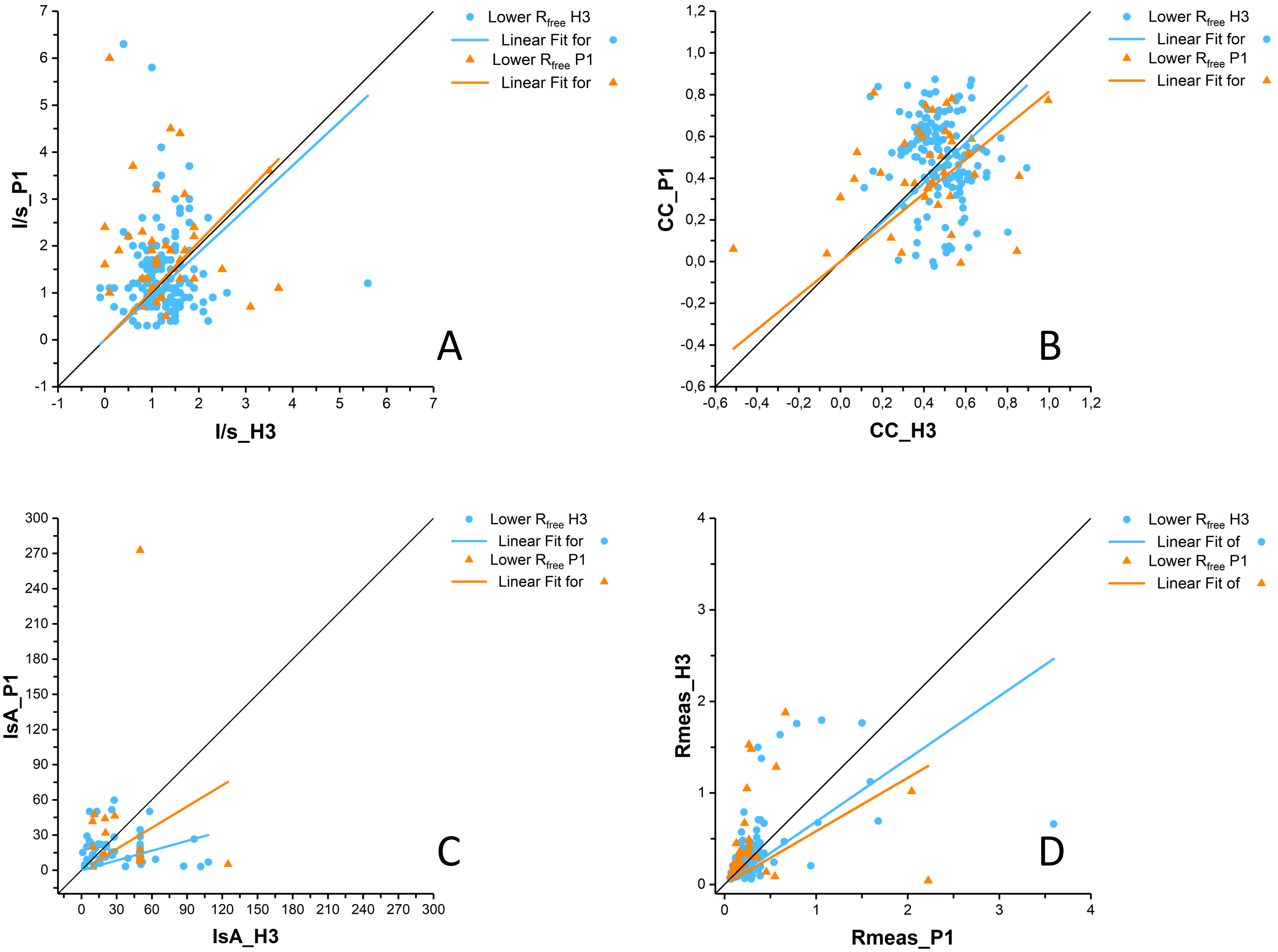

A
I/s_P1
I/s_H3
Lower R_free H3
Linear Fit for
Lower R_free P1
Linear Fit for
B
CC_P1
CC_H3
Lower R_free H3
Linear Fit for
Lower R_free P1
Linear Fit for
C
IsA_P1
IsA_H3
Lower R_free H3
Linear Fit for
Lower R_free P1
Linear Fit for
D
Rmeas_H3
Rmeas_P1
Lower R_free H3
Linear Fit of
Lower R_free P1
Linear Fit of

Supplementary Figure 1. Scatter plots of data reduction statistics for *Ct*UGGT$_{GT24}$ Fragment Based Lead Discovery datasets that could be successfully scaled both in H3 and P1, and successfully refined in either space group.

In each panel the black line is the diagonal of the plot. Datasets whose R_{free} was lower in H3 (152 datasets) are represented as cyan circles; datasets whose R_{free} was lower in P1 (38 datasets) are represented as orange triangles.

A: scatter plot of scaling I/σ(I) in the outer shell, in H3 and P1.
Linear fit (cyan circles): $^{P1}I/\sigma(I)=(0.92844 \pm 0.57205) * {}^{H3}I/\sigma(I)$ and $R^2=0.00117$;
linear fit (orange triangles): ^{P1}I/\si(I)=(1.04026 ± 0.17624)*$^{H3}I/\sigma(I)$ and $R^2=0.48496$;

B: scatter plot of scaling $CC_{1/2}$ in the outer shell, in H3 and P1.
Linear fit (cyan circles): $^{P1}CC_{1/2}=(0.94525 \pm 0.04064)*{}^{H3}CC_{1/2}$ and $R^2=0.78176$;
linear fit (orange triangles): $^{P1}CC_{1/2}=(0.81565 \pm 0.0991)*{}^{H3}CC_{1/2}$ and $R^2=0.57205$;

C: scatter plot of asymptotic $I/\sigma(I)_{asympt}$ (ISa) (Diederichs, 2010), in H3 and P1.
Linear fit (cyan circles): $^{P1}ISa=(0.27777 \pm 0.04726)*{}^{H3}ISa$ and $R^2=0.90413$;
linear fit (orange triangles): $^{P1}ISa=(0.60302 \pm 0.34239)*{}^{H3}ISa$ and $R^2= 0.66302$;

D: Log-scale scatter plot of R_{meas}, in H3 and P1.
Linear fit (cyan circles): $^{H3}R_{meas}=(0.68598 \pm 0.05418)*{}^{P1}R_{meas}$ and $R^2= 0.90413$;
linear fit (orange triangles): $^{H3}R_{meas}=(0.58206 \pm 0.15442)*{}^{P1}R_{meas}$ and $R^2 = 0.27746$.
